# ProteoformDB: an integrative database for functional roles of proteoforms

**DOI:** 10.1093/database/baag005

**Published:** 2026-02-09

**Authors:** Hanwen Luo, Sichao Qiu, Maozu Guo, Beibei Xin, Jun Wang, Guoxian Yu

**Affiliations:** School of Software, Shandong University, 1500 Shunhua Road, High-Tech Industrial Development Zone, Jinan, Shandong 250101, China; SDU-NTU Joint Centre for AI Research, Shandong University, 1500 Shunhua Road, High-Tech Industrial Development Zone, Jinan, Shandong 250101, China; School of Software, Shandong University, 1500 Shunhua Road, High-Tech Industrial Development Zone, Jinan, Shandong 250101, China; SDU-NTU Joint Centre for AI Research, Shandong University, 1500 Shunhua Road, High-Tech Industrial Development Zone, Jinan, Shandong 250101, China; Department of Computer Science, Beijing University of Civil Engineering and Architecture, No.1 Zhanlanguan Road, Xicheng District, Beijing 102616, China; Center for Crop Functional Genomics and Molecular Breeding, China Agricultural University, 2 Yuanmingyuan West Road, Haidian District, Beijing 100193, China; SDU-NTU Joint Centre for AI Research, Shandong University, 1500 Shunhua Road, High-Tech Industrial Development Zone, Jinan, Shandong 250101, China; School of Software, Shandong University, 1500 Shunhua Road, High-Tech Industrial Development Zone, Jinan, Shandong 250101, China; SDU-NTU Joint Centre for AI Research, Shandong University, 1500 Shunhua Road, High-Tech Industrial Development Zone, Jinan, Shandong 250101, China

## Abstract

Proteoforms translated from alternatively spliced transcripts contribute to the functional repertoire of the cell by performing diverse biological functions, contributing to the functional diversity of genomics and proteomics. However, the lack of existing databases that integrate functional annotations of proteoforms, and analyse the drivers of their functional differences significantly hinders in-depth research into proteoform functions. We introduce ProteoformDB, a new web resource with integrated in-platform analytical capabilities, organizes transcript-level functional annotations of proteoforms across multiple species, and provides services for prediction of proteoform functions and analysis of functional regulatory mechanisms. ProteoformDB develops user-friendly interfaces for information search, visualization, function supplement, differential analysis, and data download services. Particularly, it enables users to investigate the impact of molecular events on the function of proteoforms at multiple levels, including sequences, domains, and post-translational modifications, among others, thereby uncovering the functional differences between protein variants. The current version includes processed data (154.83 GB) for 214 animal and 28 plant species, and will become a valuable and expandable proteoform functional resource for studying genome and transcriptome functions, disease mechanisms, and other related research.

**Database URL:**  https://www.sdu-idea.cn/ProteoformDB.

## Introduction

Proteoforms are distinct protein variants derived from a single gene, resulting from mechanisms such as alternative splicing (AS), post-translational modifications (PTMs), and variations in structural domains and intrinsically disordered regions (IDRs), capturing molecular diversity at the whole-protein level, and are essential for understanding biological function [1]. Each proteoform may perform distinct roles, greatly enhancing functional diversity and complexity in genomics and proteomics [2, 3]. However, while widely present in multicellular eukaryotes, the regulatory underpinnings that govern proteoform functional specificity have not been fully elucidated [4].

Many studies show that proteoforms are closely associated with various biological processes and complex diseases. For example, early serine/arginine-rich splicing factor 2 (SRSF2) deficiency in murine endothelial cells affects the splicing pattern of several master haematopoietic regulators and significantly impairs the generation of haematopoietic stem cells, which give rise to haematopoietic diseases [5]. Another example involves alternative 5’ splice sites in BCL2L1 (commonly known as Bcl-x) pre-mRNA, which produce the anti-apoptotic Bcl-x(L) and the pro-apoptotic Bcl-x(S). Whereas Bcl-x(S) performs proapoptotic function, Bcl-x(L) exerts antiapoptotic function—and is transcriptionally up-regulated in many cancers [6]. Beyond AS, PTMs further expand the functional diversity by covalently modifying amino acid residues of mature proteoforms—such as phosphorylation, acetylation, and ubiquitination. For instance, hyperphosphorylation of Tau leads to neurofibrillary tangles in Alzheimer’s disease, whereas specific phosphorylation proteoforms of cardiac troponin I (cTni) serve as sensitive biomarkers of myocardial injury [7].

Therefore, elucidating the molecular mechanisms and functional impact of proteoforms is critical for advancing understanding of biological processes and diseases beyond the resolution of gene-level analyses, which necessitates the support of integrated analysis platforms and databases. Current research often explores potential molecular mechanisms from a single perspective and focuses on limited species. For instance, ASpedia [8] established a database for human AS annotation, enabling coordinated analysis of AS events with proteoform-specific functions. DIGGER [9] integrates information on residue-level interactions, combining domain–domain interaction (DDI) topology with protein–protein interaction (PPI) networks to systematically assess the impact of exon skipping events on proteoform structure and protein interactions. APPRIS [10] leverages an evolutionarily informed prioritization strategy, phylogenetic conservation patterns, structural–functional fingerprints, and TRIFID functional scores for predicting principal functional proteoforms of genes. HPfA [3] has constructed a standardized library of human proteoforms, providing a detailed description of human gene and protein expression patterns, while integrating proteoforms data from various tissues and cell types.

Although existing platforms and databases have considerably advanced proteoform research, they still lack a systematic integration of the cascading effects exerted by multilevel molecular events on proteoform function, leading to a fragmented understanding of the continuous process from gene to functional proteoform. Furthermore, while several resources offer cross-omics analysis capabilities [3, 11], their practical utility remains largely restricted to well-annotated organisms. Together, these limitations significantly impede progress in the study of proteoform function within biological research.

Here, we present ProteoformDB, a new web resource for investigating functional divergence among proteoforms through systematic elucidation of their molecular mechanisms. It offers unique tools for comparative and predictive analysis at the proteoform level, bridging a critical gap in current biological resources. The database integrates a user-friendly visualization interface that empowers researchers to trace the causative factors underlying proteoform heterogeneity, thereby accelerating mechanistic studies. To support large-scale cross-species analyses, ProteoformDB collects functional annotations of 4 701 580 genes across 214 animal and 28 plant species. Annual updates on 30 December ensure continuous integration of cutting-edge research, enhancing annotation accuracy while addressing emerging scientific challenges and knowledge gaps. Beyond serving as a data repository, ProteoformDB also provides an innovative, useful analysis tool for the prediction of proteoform functions, establishing an evolving resource for proteoform research.

## Materials and methods

### Framework of ProteoformDB

**Figure 1:**
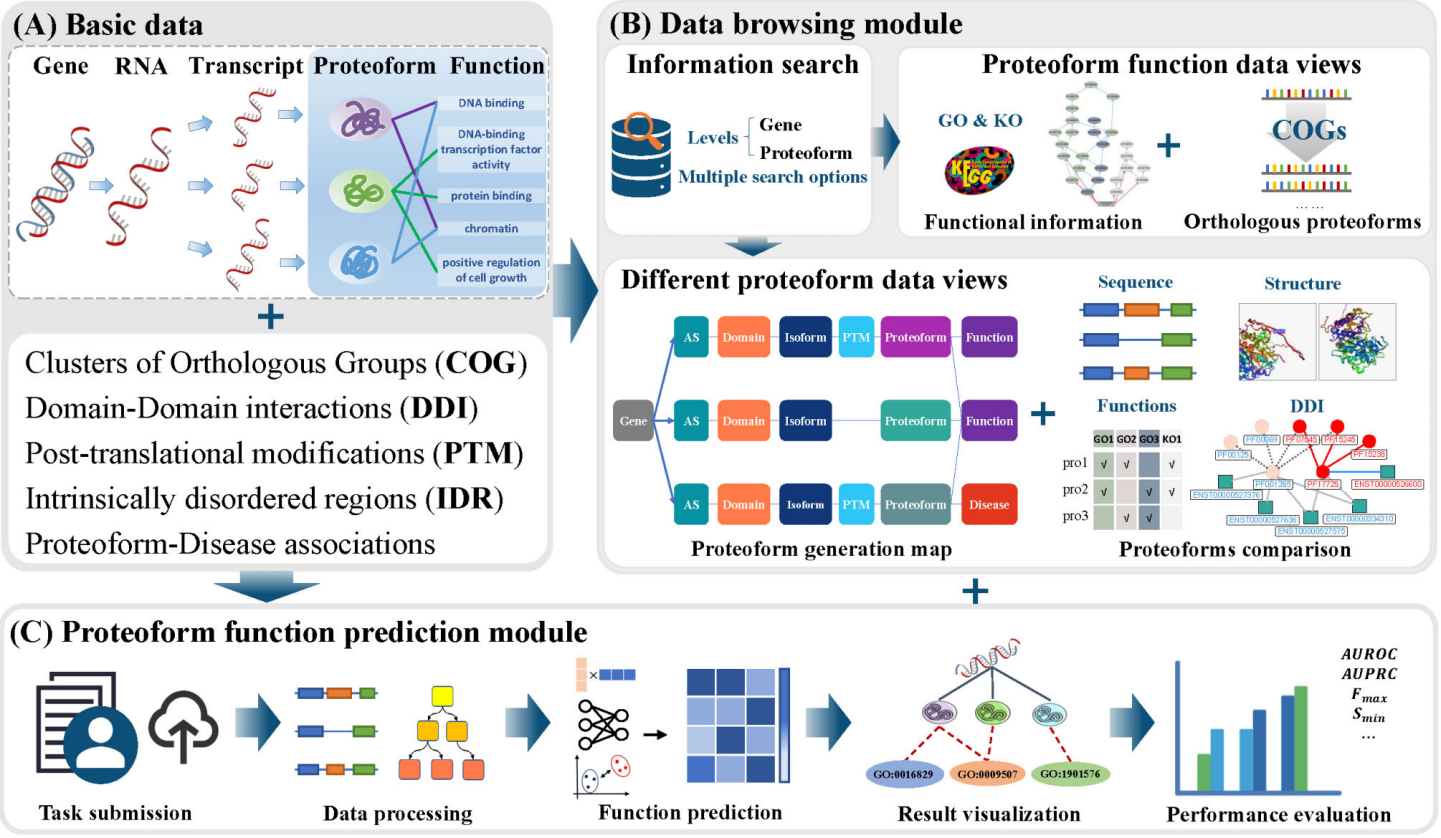
Framework overview of ProteoformDB. (A) Basic data of ProteoformDB include data from genes to proteoforms, Clusters of Orthologous Groups, domain–domain interactions, post-translational modifications, intrinsically disordered regions, and proteoform-disease associations. The data storage is powered by MySQL. (B) Data browsing module comprises five parts: information search, proteoform functional information, orthologous proteoforms search, proteoform generation map, and proteoform comparison. (C) Proteoform function prediction module implements a five-step automatic workflow, including task submission, data processing, function prediction, result visualization, and performance evaluation.

As illustrated in Fig. 1, ProteoformDB is structured around two core modules: a data browsing module and a proteoform function prediction module. The data browsing module integrates proteoform-specific molecular features—such as AS and PTMs—along with functional annotations across multiple species, providing an interactive platform for intuitive comparative analysis of underlying molecular mechanisms influencing proteoform function. The data sources are summarized in Table 1. The function prediction module employs advanced computational algorithms to infer functional annotations for specific proteoforms, addressing the current lack of web-based tools for automated proteoform function prediction. Furthermore, a detailed **Help Document** is provided, which includes comprehensive explanations of the platform’s various features and capabilities.

**Table 1:** Data sources of ProteoformDB.

Data type	Source	Link
Genome annotation	Ensembl	https://www.ensembl.org/info/data/ftp/index.html
	Ensembl Plant	http://plants.ensembl.org/info/data/ftp/index.html
Sequence	Ensembl	https://www.ensembl.org/info/data/ftp/index.html
	Ensembl Plant	http://plants.ensembl.org/info/data/ftp/index.html
GO information	GeneOntology	http://geneontology.org/docs/download-ontology
GO annotation	GOA	https://www.ebi.ac.uk/GOA/downloads.html
KO information	KEGG ORTHOLOGY	https://www.genome.jp/kegg/ko.html
KO annotation	kofamKOALA	https://www.genome.jp/tools/kofamkoala
COG annotation	eggNOG-mapper	http://eggnog-mapper.embl.de
Domain information	Ensembl	https://www.ensembl.org/info/data/ftp/index.html
	Ensembl Plant	http://plants.ensembl.org/info/data/ftp/index.html
	Interpro	https://www.ebi.ac.uk/interpro
Domain–domain interaction	DOMINE	https://manticore.niehs.nih.gov/cgi-bin/Domine?page=download
	3did	https://3did.irbbarcelona.org/download.php
	PPIDM	https://ppidm.loria.fr/download/
PTM site	dbPTM	https://awi.cuhk.edu.cn/dbPTM
IDR annotation	DisProt	https://www.disprot.org
Disease association	ASCancer Atlas	https://ngdc.cncb.ac.cn/ascancer/download

### Data integration

ProteoformDB integrates multi-omics data across genomic, transcriptomic, and proteomic levels to support a comprehensive functional understanding of proteoforms. To facilitate consistent identification and integration of heterogeneous biological data, the database uses standardized Ensembl identifiers for genes, transcripts, and proteoforms, and adopts Gene Ontology (GO) terms, KO terms, and Clusters of Orthologous Groups (COGs) for functional and pathway annotations—with COGs enabling the inference of potential functional and pathway information based on protein homology. It is important to note that the limited availability of proteoform-specific functional annotation presents a current challenge. Therefore, as a pragmatic strategy, ProteoformDB utilizes Ensembl Protein (ENSP) identifiers as proxies for proteoforms, enabling the integration of existing well-established transcript- and proteoform-level annotations while capturing molecular events that contribute to proteoform diversity.

Specifically, we systematically collect data from the Ensembl database [12], including genes, transcripts, and their encoded proteins across multiple species. Information such as chromosomal locations of genes, exon structures of transcripts, and coding sequences (including start and stop codons) was incorporated to establish a foundation for subsequent multi-source data integration.

Building on this foundation, ProteoformDB extracted proteoform sequences and employed the SUPPA2 tool [13] to identify AS events and analyse their functional consequences. To further elucidate other proteoform-specific molecular events, ProteoformDB integrated diverse structural and regulatory data, including protein domain information from Ensembl [12] and InterPro [14]; PTM sites from dbPTM [15]; and IDR annotations from DisProt [16]. For cross-dimensional data integration, ProteoformDB converted the protein coordinates of these features to genomic coordinates within coding regions—enabling accurate mapping to corresponding exons and thereby providing a comprehensive view of proteoform sequence composition, which facilitates systematic comparison of structural differences. To visually illustrate the functional implications of domain variations, ProteoformDB also incorporated DDI data from DOMINE [17], 3did [18], and PPIDM [19]. These data are presented in an interactive format, empowering users to identify potentially lost interactions and explore links to specific domains.

To support comprehensive characterization of proteoform biology, ProteoformDB integrates multi-dimensional data, including functional annotations and disease associations, following systematic identification of molecular events in proteoform transcription/translation and exploration of their functional impacts. For functional annotations, the database incorporates three core systems: it obtains standardized GO annotations (with term names, definitions, and hierarchical relationships) by mapping ENSP IDs to the GOA database [20]; it includes KO (KEGG Orthology) annotations for pathway-oriented analysis (enhancing interpretation of proteoforms in metabolic and signalling contexts) via a two-step process—first extracting proteoform sequences using Gffread [21] and SeqKit [22], then conducting functional profiling with KofamKOALA [23]; and it assigns COG [24] categories (which group proteoforms by orthology [25] to indicate conserved functions, leveraging high sequence similarity of cross-species orthologs) using eggNOG-mapper v2 [26] to boost annotation coverage and evolutionary context. Additionally, ProteoformDB integrates proteoform-disease associations curated from ASCancer Atlas [27], and harmonizes genomic coordinates via Liftover [28] to ensure consistency across genome assemblies, enabling accurate linking of disease-related AS to proteoform-specific molecular alterations.

### Difference with current databases

To provide a clearer understanding of how proteoformDB differs from other databases, we have listed relevant databases and further summarized the main distinctions in Table 2, highlighting proteoformDB’s unique advantages in analysing proteoform functional annotations and the molecular events involved in the maturation process, underscoring the comprehensive nature of proteoformDB, combining broad data integration, cross-species comparisons, multi-omics synthesis, and advanced function prediction.

**Table 2:** Comparative analysis of functional annotation capabilities across proteoform-related resources.

	Features	Cross species	Cross-omic synthesis	Function prediction
ASpedia [8]	offers a comprehensive database of functional impacts of AS events, focusing on the impacts of AS on protein functions	$\times$	$\times$	$\times$
DIGGER [9]	integrates PPI, DDI and residue-level interactions information to make exon expression analysis at a network level, advancing proteoform function research	✓	partial	$\times$
APPRIS [10]	finds the principal proteoforms of genes for a range of species based on proteoform structure, function features, and cross-species conservation	partial	partial	$\times$
HPfA [3]	provides detailed descriptions of human genes and protein expression patterns, integrates proteoform data from various tissues and cell types	$\times$	✓	$\times$
dbPTM [15]	aggregates experimentally validated PTM data while annotating potential modifications, enabling the exploration of PTM-driven proteoform functional dynamics	✓	$\times$	$\times$
neXtProt [11]	provides a comprehensive knowledgebase for human proteoforms, integrating data on protein expression, structure, function, and variants	x	✓	$\times$
Uniport [29]	serves as a central repository of proteoform sequence and functional information while providing a universal protein reference	✓	✓	$\times$
GOA [20]	provides high-quality electronic and manual GO annotations to proteins across multiple species	✓	x	$\times$
ProteoformDB	integrates multi-source data with cross-species annotations, supports high-throughput and multi-facet analysis of proteoform functions with visual mechanisms, provides the function prediction service with options	✓	✓	✓

This table highlights the key differences between ProteoformDB and existing databases/tools in supporting proteoform-level functional analysis. The cross species column indicates whether a resource supports multi-species data (✓), is limited to specific species (partial), or is species-specific (×). The cross-omic synthesis column indicates whether a resource integrates data from multiple omics levels (✓), provides limited integration (partial), or focuses on a single data type (×). The Function Prediction column indicates whether a resource supports proteoform function prediction ( denotes support, × denotes no support).

In addition, the key distinction between ProteoformDB and databases like UniProt and GOA lies in their respective research focus, analytical granularity, core functionalities, and service objectives. UniProt serves as a comprehensive universal protein knowledge base, while GOA provides a generalized functional classification and annotation framework. ProteoformDB, in contrast, is specifically designed to offer powerful tools for analysing how and why distinct proteins derived from the same gene acquire divergent functions. It provides resources for systematically understanding and predicting functional differences at the proteoform level. ProteoformDB delivers specialized capabilities for exploring the mechanistic basis of functional divergence among specific proteoforms—functionalities not provided by UniProt or GOA.

### Data browsing module

The data browsing module is composed with five parts: (i) information search, (ii) gene/proteoform information, (iii) proteoform generation map, (iv) functional similar proteoforms, and (v) proteoform comparison.

**Information search** is the interface for diverse data access. It provides a user-friendly information search portal that enables easy search of gene(s) and proteoform(s) by inputting multiple keywords, or retrieving ones with specific functional annotations by GO term ID or KO number. Batch search of multiple genes or proteoforms is also supported.

**Gene/proteoform information** establishes a visual analytics framework for multigranular functional landscapes of proteoforms. When building the GO/KO annotation view of proteoforms, GO terms annotated to proteoforms and all their ancestor terms will be displayed as a directed acyclic graph based on GO term relations, GO terms of different subontologies (BP, CC, and MF) and relations of different types (‘is a’, ‘part of’, etc.) are represented by distinct nodes and edges. All KO numbers annotated to the proteoform and their functional descriptions are provided to further comprehend proteoform functions.

**Functional similar proteoforms** part complements potential functions by transferring annotations of similar proteoforms across species. The back-end of ProteoformDB stores the association between proteoforms and COGs, enabling the transfer of functional information within the same COG. The comparison of functional differences between similar proteoforms can also be made, and all new GO and KO annotations are retrieved and emphasized as enlightening information for potential annotations. ProteoformDB further uses eggNOG-mapper tool [26] to retrieve similar proteoforms with the input query sequence.

**Proteoform comparison** part performs comparisons of function and functional factors between proteoforms. The hierarchical GO annotations diagram and function comparison table of multiple proteoforms are displayed to highlight functional differences. ProteoformDB presents the composition of various types of fragments and highlights the differential segments. It also facilitates the visualization of discrepancies in domains, PTMs, and IDRs among proteoforms. In this way, ProteoformDB can easily identify differential sequence fragments, domains, PTMs, and IDRs between proteoforms and thus to find out factors that cause the functional difference.

**Proteoform generation map** employs hierarchical visualization techniques to elucidate the continuum from a gene to its proteoforms. By layering biological dimensions, it dynamically maps molecular events, including AS, PTM, and IDR, onto an interactive multi-layered map. Moreover, to assist in disease research, it further integrates proteoform-disease association information. To balance information density and interface simplicity, it incorporates a synchronized panel. When users focusing on a specific proteoform, the panel will display its detailed generation process. Conversely, when aiming to analyse the sources of functional differences, it can select nodes of GO terms or disease associations, enabling ontology-based dynamic comparison of developmental trajectories among proteoforms and linking proteoform functionalities to underlying molecular events. This design empowers users to dissect heterogeneity origins (e.g. exon skipping and synergizing with IDR phase transitions) while preserving a holographic causality framework from gene to functional divergence.

In addition to the aforementioned core functions, the data browsing model provides the **Data Download** function, enabling users to access organized annotation data for each species to conduct large-scale research and analysis. Users can conveniently choose specific species to perform bulk downloads of relevant functional information, including GO and KO annotations, COG classifications, and PTM data. This feature streamlines the data acquisition process, allowing researchers to efficiently gather comprehensive datasets tailored to their specific needs. In addition, the retrieved and predicted results in the following parts can be easily downloaded in their respective pages.

### Proteoform function prediction module

Proteoform function prediction is critical for deciphering how proteoform molecular differences drive biological function, yet existing tools have clear limitations in this task: visualization tools like Protter [30] only display proteoform features (e.g. domains, PTMs) without functional inference, while services like PredictProtein [31] focus more on general sequence/structure prediction rather than targeting proteoform-specific functional differences.

To address this gap, ProteoformDB provides the dedicated proteoform function prediction module. As shown in Fig. 1C, users can predict the proteoform function on a workflow with five steps: (i) task submission, (ii) data preprocess, (iii) function prediction, (iv) result visualization, and (v) performance evaluation.

**Task submission** step encompasses task configuration, submission, and schedule management. Users can select the desired prediction model, configure parameters and upload data to the server, the tool performs a file format check to ensure that the files uploaded by users can be legally input into the model.

**Data preprocess** step is responsible for automatically processing and formatting the data uploaded by users to meet the data requirements of the selected prediction model. The tool formats the raw data and utilizes the *k*-mer to encode features of proteoforms. To enhance the prediction efficiency, a pre-processing strategy is implemented that matches the necessary GO terms and extracts relevant data, including hierarchical relationships and feature representations, for input into the prediction model.

**Function prediction** step assembles four methods that have been widely studied as competitive baselines to differentiate proteoform functions [32–36]: (i) IsoFun constructs a heterogeneous network from expression and interaction data and applies bi-random walks to infer proteoform—GO associations [37]; (ii) DisoFun collaboratively factorizes proteoform and gene-term matrices to identify functional proteoforms while incorporating PPI and GO structures [38]; (iii) IsoResolve utilizes domain adaptation to align gene-level and proteoform-level feature distributions, facilitating knowledge transfer from genes to proteoforms [39]; (iv) IsofunGO employs hierarchical GO network embedding to compress ontological annotations and an attention-based MIL network to integrate multi-omics features for proteoform function prediction [40]. These methods are applicable to various data scenarios, as shown in Table 3.

**Table 3:** Comparison of data requirements for proteoform function prediction methods.

	IsoFun	DisoFun	IsoResolve	IsofunGO
RNA-seq data	✓	✓	✓	✓
Sequence	–	–	–	✓
Gene annotation	✓	✓	✓	✓
GO hierarchy	✓	✓	–	✓
GGI network	✓	✓	–	–

**Result visualization** step constructs a diagram to illustrate gene-proteoform relationships and predicted associations with GO terms. This visualization provides users with an intuitive understanding of the data and results.

**Performance evaluation** is conducted at both the gene and proteoform levels to comprehensively assess prediction accuracy. At the proteoform level, Area Under the Receiver Operating Characteristic curve (AUROC) and Area Under the Precision-Recall Curve (AUPRC) are computed by treating each GO term prediction as a separate binary classification task, using the predicted confidence scores and available experimental annotations per proteoform. For gene-level evaluation, predictions from all proteoforms of a gene are aggregated by selecting the maximum prediction score per GO term, and performance metrics are calculated against gene-level ground truth annotations.

## Result

### Information search in ProteoformDB

**Figure 2:**
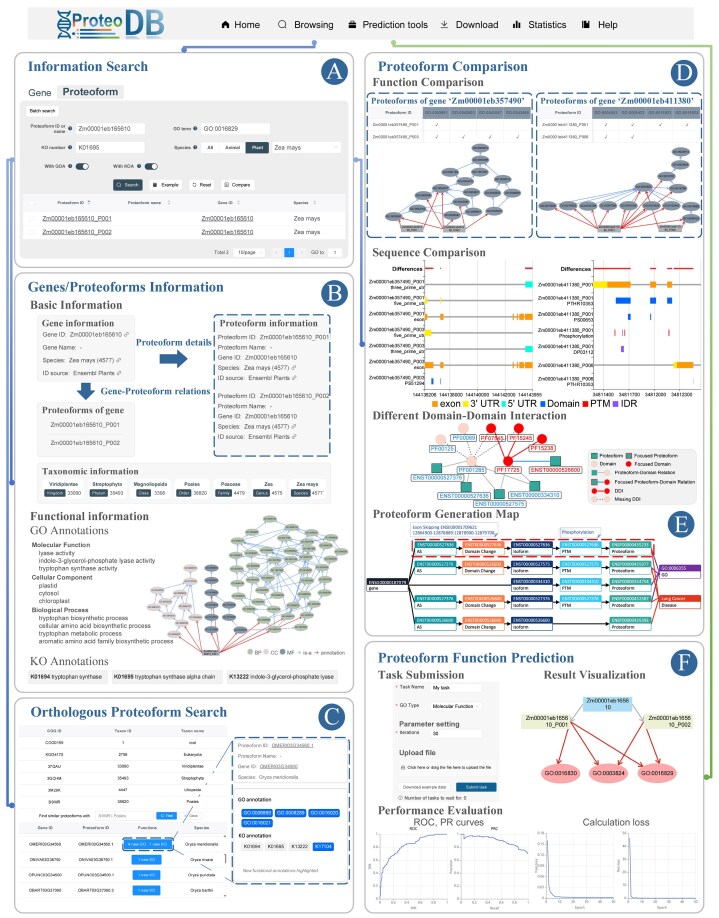
Usage of ProteoformDB. (A) On the ‘Information Search’ page, users can perform the search of proteoforms and genes through multiple keywords search or batch search. The search results provide access to gene and proteoform information pages, allowing users to explore detailed information. Additionally, users can select multiple proteoforms for comparison. (B) The ‘Gene/Proteoform information’ page offers both basic and functional information about genes and their corresponding proteoforms. The GO and KO annotations of proteoforms are displayed using a visual and interactive component, enhancing the understanding of their functional characteristics. (C) The ‘Orthologous Proteoform Search’ page allows users to discover functionally similar proteoforms across different species within the same COG. Newly identified functional annotations are highlighted, providing valuable insights into proteoform functions. (D) The ‘Proteoform Comparison’ page enables users to identify differences between proteoforms from functions and multiple functional factors. It assists users to pick out specific factors of sequence fragments, domains, PTMs, and IDRs that cause functional variations between proteoforms. The DDI part displays the impact of DDI on diverse proteoforms. (E) The proteoform generation map elucidates the intricate continuum from a gene to its proteoforms, it also unveils pivotal factors associated with the functional significance and diseases of these proteoforms. (F) The ‘Proteoform Function Prediction’ page enables users to submit proteoform function prediction tasks and present results using visualizations and various evaluation metrics.

ProteoformDB offers a user-friendly search tool for retrieving gene and proteoform information. For example, when searching proteoforms of the maize gene ‘Zm00001eb165610’, users find that ‘Zm00001eb165610_P001’ catalyses the formation of free indole, while ‘Zm00001eb165610_P002’ is involved in the synthesis of L-tryptophan from indole-3-glycerol phosphate [29, 41]. Figure 2A shows the search interface, allowing users to browse gene and proteoform details in the results. The basic information of gene ‘Zm00001eb165610’ and its two proteoforms is displayed in Fig. 2B, where users can explore GO and KO annotations. For example, ‘Zm00001eb165610_P001’ is annotated with 10 specific GO terms and 3 KOs, including indole-3-glycerol-phosphate lyase activity (GO:0033984), while Zm00001eb165610_P002 shares ancestor terms of these GO annotations.

### Functional similar proteoforms

A clade list of COGs, ordered from broadest to narrowest, is presented (Fig. 2C). The COG information for ‘Zm00001eb165610_P001’ spans five ranks, from Eukaryota to Poales. For instance, at the Poales rank, a proteoform from the species Oryza meridionalis has four newly annotated GO terms and three new KO annotations, suggesting that ‘Zm00001eb165610_P001’ may share these unexplored functions.

### Comparison of proteoforms

By selecting multiple proteoforms from the search results, users can enter the comparison interface. For functional comparisons, GO annotation relationships of all proteoforms are integrated into a GO-DAG visual component. Differences in functional annotations are presented in a comparison table, allowing users to examine the diverse aspects of proteoforms from various data views. Additionally, comparisons of proteoform functions and sequences within or across genes can be conveniently performed on the gene information page.

Figure 2D illustrates the comparison of proteoforms from two maize genes, ‘Zm00001eb357490’ and ‘Zm00001eb411380’. For ‘Zm00001eb357490’, ‘Zm00001eb357490_P003’ is the most conserved and expressed variant and exhibits specific DNA-binding activity, while ‘Zm00001eb357490_P001’ lacks this specificity. This functional divergence is linked to differences in exon structure between their corresponding transcripts, leading to variations in proteoform domains, including an additional Myb domain in ‘Zm00001eb357490_P003’ that enhances DNA-binding capability. Similarly, for ‘Zm0000-1eb411380’, ‘Zm00001eb411380_P001’, and ‘Zm00001eb411380_P006’ show differing enzymatic activities, with ‘Zm00001eb411380_P001’ catalysing multiple reactions and ‘Zm00001eb411380_P006’ limited to beta-glucosidase activity. These differences arise from variations in exon composition and domain structures, with additional exons in ‘Zm00001eb411380_T001’ contributing to a broader range of catalytic activities and the presence of phosphorylation sites and IDRs that influence enzyme function and regulatory mechanisms.

In another example, the ‘ENSP00000435233’ derived from the human gene ‘ENSG00000187079’ functions as a transcription factor critical to the Hippo signalling pathway, which regulates organ size and suppresses tumors by controlling cell proliferation and apoptosis [29]. Figure 2E highlights that this proteoform results from an exon-skipping AS event (ENSE00001709621), which is linked to several cancers, including lung, breast, and ovarian cancer [27]. The proteoform data views from ProteoformDB reveal differences in proteoform generation pathways and identify key AS events and domain variations associated with these cancers. Additionally, comparing domains and PTMs among proteoforms of ‘ENSG00000187079’ related to the function regulation of DNA template translation (GO:0006355) shows that functional proteoforms possess an additional PF01285 domain and exhibit specific phosphorylation modifications. Studies suggest that the absence of this domain and phosphorylation may impair transcriptional regulation function [42].

### Proteoform function prediction

As shown in the task submission part of Fig. 2F, users can select the prediction method and its required data. Users can upload data and use default or update input parameters to submit tasks. The status and progress of submitted tasks are displayed in real-time, once the tasks are completed, users can view the prediction results and performance evaluation, the prediction results are packaged for users to download.

In the result visualization part of Fig. 2F, take the prediction results of two ‘Zm00001eb165610_P001’ and ‘Zm00001eb165610_P002’ generated from maize gene ‘Zm000-01eb165610’ as an example. While ‘Zm00001-eb165610_P001’ is additionally annotated with ‘carbon–carbon lyase activity’ (GO:0016830). These two proteoforms have different catalytic functions; the former catalyses the formation of free indole from indole-3-glycerol phosphate [41], while the latter catalyses the formation of L-tryptophan [29]. The indole-3-glycerol-phosphate lyase is a specific enzyme in the carbon–carbon lyase family, while tryptophan synthase is not. The prediction tool precisely differentiates the functions of two proteoforms generated from the same gene. The evaluation part of Fig. 2F reports the performance of prediction results, ROC and PR curves of ‘catalytic activity’, and the loss change during the training process.

### Performance of proteoform function prediction

ProteoformDB integrates multiple algorithms to accommodate diverse user requirements regarding input data types, accuracy, and efficiency. We evaluated algorithm performance on human (7657 genes; 27 777 proteoforms) and maize (8732 genes; 17 157 proteoforms) datasets at gene/proteoform levels, covering 4108 and 1872 GO terms, respectively.

Table 4 (evaluated by AUROC/AUPRC) shows that IsofunGO outperforms the other algorithms in most instances, and this is because IsofunGO fully utilizes information-rich sequence information and simultaneously models the GO hierarchy to embed GO terms and efficiently compress massive GO annotations. In addition, IsofunGO has the capability to resist the impact of imbalance of functional annotation data. Although IsoFun and DisoFun also utilize GO hierarchy, they mainly capture linear relationship between proteoforms and GO terms, resulting in relatively lower performance. Similarly, IsoResolve does not leverage sequence information, resulting in comparatively lower performance than IsofunGO. Notably, low AUPRC values across methods reflect extreme label imbalance inherent to GO terms.

**Table 4:** Performance of integrated function prediction methods on human and maize datasets.

		Human				Maize			
		Gene-level		Proteoform-level		Gene-level		Proteoform-level	
GO	Algorithm	AUROC	AUPRC	AUROC	AUPRC	AUROC	AUPRC	AUROC	AUPRC
BP	IsoFun [37]	0.556	0.011	0.550	0.007	0.649	0.028	0.619	0.027
	DisoFun [38]	0.538	0.023	0.601	0.017	0.584	0.027	0.509	0.020
	IsoResolve [39]	0.651	0.021	0.607	0.012	0.661	0.032	0.627	0.020
	IsofunGO [40]	0.656	0.072	0.662	0.057	0.920	0.681	0.917	0.670
CC	IsoFun [37]	0.584	0.011	0.574	0.006	0.552	0.019	0.554	0.027
	DisoFun [38]	0.625	0.021	0.621	0.013	0.559	0.060	0.497	0.050
	IsoResolve [39]	0.659	0.028	0.603	0.018	0.657	0.070	0.652	0.054
	IsofunGO [40]	0.696	0.130	0.698	0.121	0.828	0.490	0.805	0.455
MF	IsoFun [37]	0.536	0.015	0.538	0.009	0.680	0.029	0.660	0.025
	DisoFun [38]	0.553	0.020	0.623	0.014	0.556	0.021	0.546	0.015
	IsoResolve [39]	0.617	0.019	0.590	0.014	0.662	0.025	0.643	0.021
	IsofunGO [40]	0.777	0.172	0.768	0.150	0.936	0.629	0.932	0.611

Besides, we compared the runtime of different algorithms, the runtime of proteoform function prediction algorithms is greatly related to the number of genes and labels. Thus, we conducted runtime tests on datasets with different numbers of genes and labels. The results are shown in Table 5, where *N* and *M* are the number of genes and labels, respectively. The runtime includes the total time of data processing and algorithm operation. As the number of genes and labels increases, the algorithm runs slower. DisoFun achieves the shortest runtime due to its efficient matrix decomposition method. IsoFun has a high runtime, as it requires more processing time to construct heterogeneous networks during the data processing phase. The runtime of IsofunGO is relatively stable with changes in the number of labels, due to the GO embedding and massive GO annotations compressing conducted by IsofunGO. IsoResolve performs multiple binary classification tasks to predict the functions of isoforms, so its runtime is greatly impacted by the label scale.

**Table 5:** The runtime of integrated function prediction algorithms with different numbers of genes (*N*) and labels (*M*).

		*M*		
		100	500	1000
IsoFun	*N* = 2000	222s	222s	223s
	*N* = 4000	1289s	1292s	1353s
	*N* = 6000	3930s	3931s	3902s
DisoFun	*N* = 2000	39s	39s	40s
	*N* = 4000	117s	118s	148s
	*N* = 6000	299s	301s	332s
IsoResolve	*N* = 2000	39s	175s	361s
	*N* = 4000	122s	479s	927s
	*N* = 6000	240s	951s	1813s
IsofunGO	*N* = 2000	287s	265s	265s
	*N* = 4000	823s	820s	848s
	*N* = 6000	1695s	1702s	1689s

We further conducted some case studies on human and maize data. Particularly, we collected 8 genes and 21 proteoforms with known annotations, which are related to ‘DNA-binding transcription factor activity’ (GO:0003700), ‘GTPase activity’ (GO:0003924), ‘serine-type endopeptidase activity’ (GO:0004252), ‘signalling receptor binding’ (GO:0005102), ‘endopeptidase activity’ (GO:0004175), ‘oxidoreductase activity, acting on paired donors, with oxidation of a pair of donors resulting in the reduction of molecular oxygen to two molecules of water’ (GO:0016717), ‘RNA nuclease activity’ (GO:0004540), and ‘xyloglucan: xyloglucosyl transferase activity’ (GO:0016762) [12, 29]. The known and predicted annotations are presented in Tables 6 and 7. These experimental results demonstrate the usability and effectiveness of ProteoformDB in proteoform function prediction tasks. Although IsofunGO requires more input data types, it achieves a more accurate proteoform function prediction and can distinguish functional differences in proteoforms of the same gene, thus meeting the high-accuracy prediction demands of users. IsoResolve can identify the function of proteoform, but it has limitations in distinguishing the functions of proteoforms spliced from the same gene. However, its flexibility in input data types facilitates its broad applicability. DisoFun and IsoFun offer comparatively lower performance, but DisoFun excels in efficiency when handling large datasets. As for IsoFun, it constructs a heterogeneous network that includes genes, proteoforms, and GO terms to model rich relationships between them, making it more interpretable in function prediction. Therefore, ProteoformDB integrates these algorithms as optional choices for users.

**Table 6:** The known annotations (fourth column) and predicted positive/negative (✓/$\times$) annotations of proteoforms on human data.

GO	Gene	Proteoform	Annotation	IsofunGO	IsoResolve	DisoFun	IsoFun
GO:0003700	ENSG00000184271	ENSP00000448389	✓	✓	✓	✓	$\times$
		ENSP00000330190	$\times$	$\times$	✓	$\times$	$\times$
GO:0003924	ENSG00000166592	ENSP00000462559	✓	✓	$\times$	$\times$	$\times$
		ENSP00000461995	$\times$	$\times$	✓	✓	$\times$
GO:0004252	ENSG00000100263	ENSP00000384113	✓	$\times$	✓	$\times$	$\times$
		ENSP00000413128	✓	$\times$	✓	✓	✓
		ENSP00000399550	$\times$	$\times$	✓	$\times$	✓
GO:0005102	ENSG00000125378	ENSP00000453365	✓	✓	$\times$	$\times$	$\times$
		ENSP00000453691	$\times$	$\times$	$\times$	$\times$	$\times$
		ENSP00000453467	$\times$	$\times$	✓	$\times$	$\times$
GO:0004175	ENSG00000100263	ENSP00000384113	✓	$\times$	✓	$\times$	$\times$
		ENSP00000399550	$\times$	$\times$	✓	$\times$	✓
		ENSP00000413128	✓	✓	✓	✓	✓

**Table 7:** The known annotations (fourth column) and predicted positive/negative (✓/$\times$) annotations of proteoforms on maize data.

GO	Gene	Proteoform	Annotation	IsofunGO	IsoResolve	DisoFun	IsoFun
GO:0016717	Zm00001eb409690	Zm00001eb409690_P001	✓	✓	$\times$	$\times$	$\times$
		Zm00001eb409690_P003	$\times$	$\times$	✓	✓	$\times$
GO:0004540	Zm00001eb320100	Zm00001eb320100_P001	✓	$\times$	✓	✓	$\times$
		Zm00001eb320100_P002	$\times$	$\times$	✓	$\times$	✓
GO:0016762	Zm00001eb071640	Zm00001eb071640_P001	✓	✓	✓	$\times$	✓
		Zm00001eb071640_P002	$\times$	✓	$\times$	$\times$	$\times$
		Zm00001eb071640_P003	✓	✓	✓	$\times$	$\times$
		Zm00001eb071640_P004	✓	✓	✓	$\times$	✓

### Prevalence of functional divergence across species

**Figure 3:**
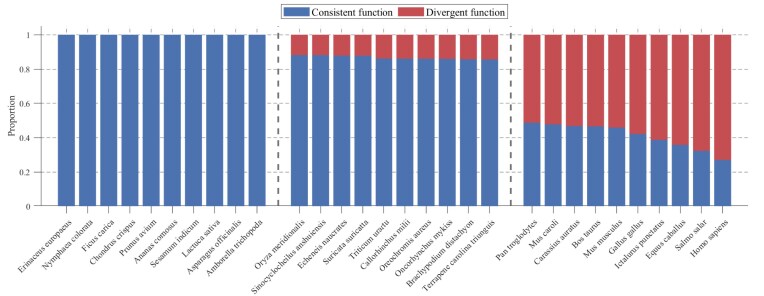
Functional consistency and divergence profiles across species. The bar plot displays the proportion of proteoforms of genes exhibiting consistent versus divergent functions for 30 representative species selected from the extremities and mid-range of the functional divergence spectrum. Species are grouped by divergence rank (low, medium, and high divergence ratios) with vertical dashed lines separating the groups.

Based on the comprehensive analysis of functional consistency across 200+ species in ProteoformDB, we quantified the proportion of genes exhibiting functional divergence among their proteoforms, as shown in Fig. 3. Applying the criteria (proteoforms with one functionally annotated proteoform; taxa with 200 qualifying genes), we analysed proteoform-level functional divergence across 242 species using the data integrated in ProteoformDB. The proportion of genes exhibiting functional divergence among their proteoforms varies substantially across species. Homo sapiens shows particularly pronounced divergence, with 73.04% of genes encoding proteoforms with distinct functional annotations. Other mammals also exhibit high divergence rates (Pan troglodytes: 51.30%; Bos taurus: 53.45%), while certain fish species such as Astatotilapia calliptera (34.65%) and model organisms including Danio rerio (40.55%) demonstrate significant but variable levels of functional divergence. Even in species with relatively higher functional consistency, such as Phaseolus vulgaris (1.58%) and Arabidopsis thaliana (22.26%), the presence of divergent proteoforms remains biologically substantial. The substantial interspecies variation in divergence rates highlights the necessity of investigating gene function at the proteoform level. Notably, it is important to acknowledge that the observed differences in functional divergence across species may be substantially influenced by technical and annotation-related factors, including disparities in research depth—such as variations in functional annotation completeness—database annotation biases or inconsistencies, species-specific investigation priorities, and technical limitations in the detection and annotation of certain proteoforms. In particular, the notably high level of functional divergence observed in mammals (e.g. humans) may reflect a disproportionate amount of research attention, leading to more comprehensive functional annotation and thus higher detection of proteoform-level functional differences. Furthermore, evolutionary proximity and annotation transfer practices—where proteoform annotations from well-studied species are applied to closely related organisms—may also contribute to the observed patterns, independent of actual biological divergence.

## Discussion

Proteoforms, generated through mechanisms such as AS and PTMs, play a central role in expanding functional diversity within biological systems. These proteoforms are closely linked to critical biological processes and diseases, including cancer and other complex disorders. Despite the growing recognition of proteoforms’ significance, there has been a gap in providing a comprehensive, integrative platform for their functional analysis. Our newly developed database, ProteoformDB, is designed to address this gap by offering detailed functional annotations and comparison tools for proteoforms across multiple species. ProteoformDB enhances the understanding of proteoform functions by integrating GO, KO, and COG annotations, enabling cross-species comparisons and supporting functional annotation prediction. With visual and interactive components, researchers can easily explore key aspects such as AS events, domain variations, and PTMs that contribute to functional differences and disease associations. By providing a scalable resource currently covering 242 species, ProteoformDB serves as a foundational tool for future research in areas ranging from genomics to disease pathology. We anticipate expanding the database to include proteoform-disease association data, further enhancing its utility for biomedical research. ProteoformDB represents a significant step towards elucidating genome function at the proteoform level, thereby advancing the fields of biology.

Despite its extensive coverage and integrated multi-level architecture, ProteoformDB still reflects several challenges currently faced in the field of proteoform research. A major issue is the limited availability of functional annotations at the proteoform level. This constraint reduces the granularity of functional analysis possible for proteoforms and necessitates reliance on transcript-level data as a practical compromise. Although this approach allows the integration of functional information from both transcript and protein levels, it falls short of fully capturing the distinct functional roles that may be associated with each specific proteoform.

Furthermore, ProteoformDB currently focuses on proteoform diversity originating from multiple molecular mechanisms within a reference genome framework. However, it does not yet incorporate genetic variation (e.g. single nucleotide polymorphisms, SNPs; and insertions and deletions, indels) or functional evidence from gene knockout or site-directed mutagenesis studies, which are critical for understanding individual- or population-specific proteoform functions and their roles in disease and represent an important direction for future development, as advances in proteogenomics and functional genomics increasingly enable proteoform-resolved annotation at population scale.

In addition, the current version lacks a fully implemented feature for transparent data verification—direct links to original experimental publications (via PubMed ID or DOI). This enhancement is a high-priority item in our development roadmap. We plan to curate primary literature references from integrated sources and provide accessible links on proteoform information pages in future releases.

## Data Availability

ProteoformDB is publicly available at https://www.sdu-idea.cn/ProteoformDB, without the need of registration or login.
